# Prevalence of Insulin Resistance in the Hungarian General and Roma Populations as Defined by Using Data Generated in a Complex Health (Interview and Examination) Survey

**DOI:** 10.3390/ijerph17134833

**Published:** 2020-07-04

**Authors:** Róza Ádány, Péter Pikó, Szilvia Fiatal, Zsigmond Kósa, János Sándor, Éva Bíró, Karolina Kósa, György Paragh, Éva Bácsné Bába, Ilona Veres-Balajti, Klára Bíró, Orsolya Varga, Margit Balázs

**Affiliations:** 1 MTA-DE Public Health Research Group, Public Health Research Institute, University of Debrecen, 4032 Debrecen, Hungary; piko.peter@sph.unideb.hu (P.P.); balazs.margit@sph.unideb.hu (M.B.); 2 Department of Preventive Medicine, Faculty of Public Health, University of Debrecen, 4032 Debrecen, Hungary; fiatal.szilvia@sph.unideb.hu (S.F.); sandor.janos@sph.unideb.hu (J.S.); biro.eva@sph.unideb.hu (E.B.); varga.orsolya@sph.unideb.hu (O.V.); 3 Department of Health Methodology and Public Health, Faculty of Health, University of Debrecen, 4400 Nyíregyháza, Hungary; kosa.zsigmond@foh.unideb.hu; 4 Institute of Behavioural Sciences, Faculty of Public Health, University of Debrecen, 4032 Debrecen, Hungary; kosa.karolina@sph.unideb.hu; 5 Institute of Internal Medicine, Faculty of Medicine, University of Debrecen, 4032 Debrecen, Hungary; paragh.gyorgy@med.unideb.hu; 6 Institute of Sport Management, University of Debrecen, 4032 Debrecen, Hungary; bacsne.baba.eva@econ.unideb.hu; 7 Department of Physiotherapy, Faculty of Public Health, University of Debrecen, 4032 Debrecen, Hungary; balajti.ilona@sph.unideb.hu; 8 Department of Health Systems Management and Quality Management in Health Care, Faculty of Public Health, University of Debrecen, 4032 Debrecen, Hungary; kbiro@med.unideb.hu

**Keywords:** Roma population, health survey, metabolic syndrome, insulin resistance, cut-off values for surrogate indices, HOMA-IR, QUICKI, McAuley index, TG/HDL-C ratio, TyG index

## Abstract

Data mainly from one-off surveys clearly show that the health of Roma, the largest ethnic minority of Europe, is much worse than that of the general population. However, results from comprehensive exploratory studies are missing. The aim of our study was to create a complex database for comparative and association studies to better understand the background of the very unfavourable health of Roma, especially the high burden of cardiometabolic diseases. A three-pillar (questionnaire-based, physical and laboratory examinations) health survey was carried out on randomly selected samples of the Hungarian general (HG, *n* = 417) and Roma (HR, *n* = 415) populations, and a database consisting of more than half a million datapoints was created. Using selected data, the prevalence rates of metabolic syndrome (MetS) and of its components were determined, and to estimate the risk of insulin resistance (IR), surrogate measures (the homeostasis model assessment of insulin resistance index, quantitative insulin sensitivity check index, McAuley and TyG indices and the TG/HDL-C ratio) were calculated. Receiver operating characteristic curve analysis and Youden’s method were used to define the optimal cut-off values of each IR index. The prevalence of MetS was very high in both study populations (HG: 39.8%, HR: 44.0%) with no statistically significant difference between the two groups in females or males. The prevalence of MetS showed a very marked increase in the HR 35–49 years age group. Among surrogate measures, the TyG index showed the greatest power for predicting IR/MetS at a cut-off value of 4.69 (77% sensitivity, 84% specificity) and indicated a 42.3% (HG) and 40.5% (HR) prevalence of IR. The prevalence of MetS and IR is almost equally very unfavourable in both groups; thus, the factors underlying the high premature mortality burden of Roma should be further clarified by investigating the full spectrum of risk factors available in the database, with a special focus on the access of Roma people to preventive and curative health services.

## 1. Introduction

Roma are the largest ethnic minority population in Europe, with an estimated population of 10–12 million [1]. Although restrictions exist on collecting statistical data on health and its determinants by ethnic status in the countries where Roma populations are most concentrated (Southern, Central, and Eastern European countries) [2,3], increasing amounts of data from ethically appropriate one-off surveys are available and show that the health of Roma is much worse than that of the general population [1]. These studies are almost exclusively descriptive ones on the prevalence of certain diseases, especially infectious and certain genetic diseases [1,4,5], and of health determinants [6,7,8,9], especially cardiovascular risk factors [10,11,12], while comprehensive exploratory studies are missing.

In addition, common study limitations include a low number of participants and the absence of a majority population group as a reference. For example, the latest literature review on the prevalence of diabetes mellitus among Roma notes that altogether, four previously published papers suggested a higher prevalence of diabetes among Roma than in Caucasians, and “none of them reached the standards regarding representative samples and number of cases for a conclusive result” [13].

Although the relationship between the very unfavourable socio-economic conditions and poor health status among Roma [14,15,16] is almost evident, the differences observed in comparisons between the Roma population and the general population do not appear to be explained entirely by their worse socio-economic status [17,18]. Recent studies on the genetic background of the increased risk of different non-communicable diseases among Roma [19,20,21] further strengthen the hypothesis that the health status of Roma is defined by a complexity of different health-related factors.

Metabolic syndrome (MetS) is considered to be the most robust predictor of the increased susceptibility to different non-communicable diseases (cardiovascular diseases, type 2 diabetes, polycystic ovary syndrome, fatty liver, cholesterol gallstones, asthma, sleep disturbances, and some forms of cancer) [22], most of which have a high morbidity and mortality burden especially among vulnerable populations [23]. Insulin resistance (IR) defined as an impaired biological response to insulin actions in the insulin-responsive tissues is considered central to the pathology of MetS. IR can be developed by different mechanisms most commonly initiated by lifestyle factors (unhealthy diet, smoking, physical inactivity) and resulting in hyperglycaemia and hyperinsulinemia in its early phase and the affected subjects subsequently develop multiple metabolic disturbances [24,25,26]. The link between insulin resistance (IR) and MetS has long been well known [27,28], and increasing numbers of studies have demonstrated that IR is the root cause of clustering disturbances in glycose and lipid metabolisms in different target tissues (muscle, adipose tissue, liver), resulting in not only hyperglycaemia, but also elevated TG, decreased HDL-C, and a typically central type of obesity [29,30,31,32]. In addition, abundant clinical and epidemiologic evidence has demonstrated a close linkage between IR and hypertension via enhanced salt absorption in the kidney, activation of the sympathetic nervous system, and alteration in vascular resistance (see reviewed [33]). Findings suggest that IR is significantly associated with the clustering of MetS risk factors [34,35,36,37], and IR is considered central to the pathophysiology of cardiometabolic diseases.

The study we present here is the most complex combined survey ever carried out using mixed methodology among Roma to not only determine the distribution of different health conditions, risk factors, and health determinants in comparison with those of the general population living in their neighbourhood, but also to create a database that can be used for association studies to explore the interactions between different environmental and genetic factors in defining health status and susceptibility to different diseases.

In the present paper, in addition to providing a detailed description of the methodology used to create a complex database and to introduce its composition, we demonstrate its usefulness by first describing how it can be used to examine the prevalence of cardiometabolic risk factors linked to insulin resistance after defining the cut-off points for different IR surrogate indices for metabolic syndrome and its prevalence among Roma in comparison with the general population.

## 2. Materials and Methods

### 2.1. Sample Representative of the Hungarian Roma (HR) Population Living in Segregated Colonies in Northeast Hungary

**Figure 1:**
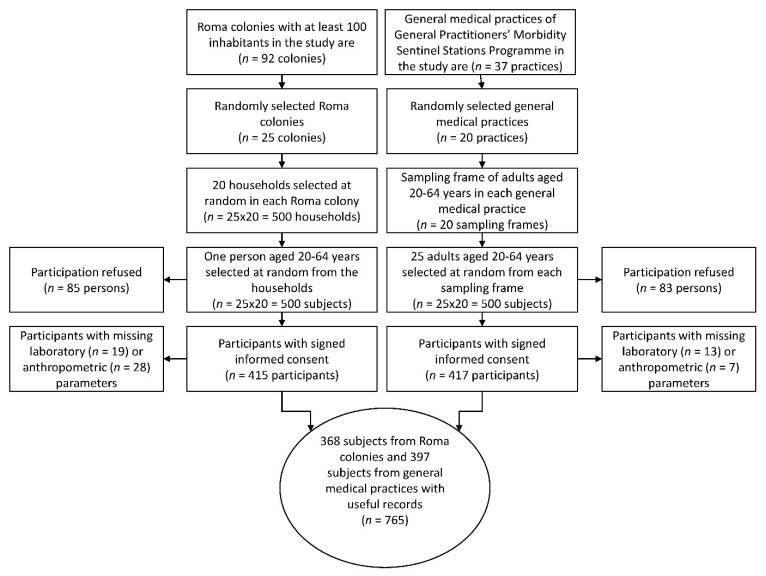
Flowchart showing the process of sample selection for study populations.

The study population was enrolled from two counties (Hajdú-Bihar and Szabolcs-Szatmár-Bereg) in Northeast Hungary, the area where the Roma are most prevalent and where the majority of segregated Roma colonies are located. Ninety-two segregated colonies identified previously in a nationwide survey with more than 100 inhabitants [38] were considered in the study, of which 25 were randomly selected using general practitioners’ (GPs) validated household lists. Afterwards, 20 households in each colony were randomly chosen, and one person aged 20–64 years from each household was interviewed face-to-face at the respondent’s household by Roma university students under the supervision of public health coordinators and was invited to visit a general practitioner for a physical examination and blood collection. The ethnicity of the participants was assessed by self-declaration. The planned sample size was 500 individuals, and among them, 415 committed to participate in the study (Figure 1).

### 2.2. Sample Representative of the Hungarian General (HG) Population Living in Northeast Hungary

The control study population involved randomly drawn individuals who were 20 to 64 years of age, lived in private households in the same counties of Northeast Hungary and were registered by general practitioners involved in the General Practitioners’ Morbidity Sentinel Stations Programme (GPMSSP). The GPMSSP is a population-based registry that was established in 1998 to monitor the prevalence and incidence of chronic non-communicable diseases of great public health importance [39]. From 20 randomly selected GP practices 25 individuals selected at random/practice were invited to participate in the study. For subjects who could not be reached, other individuals were enrolled, but if someone refused to participate, drawing another person was not allowed. The planned sample size was 500 individuals, and among them 417 could be enrolled into the study (Figure 1).

### 2.3. Pillars of the Complex (Health Interview and Examination) Survey

A three-pillar survey consisting of questionnaire-based interviews (I), physical examinations (II) and laboratory tests (III) was designed and carried out.

#### 2.3.1. I. Questionnaire-Based Interviews

The main part of the questionnaire in our survey was the European Health Interview Survey (EHIS) wave 2 (EHIS 2 for 2013–2015, used in the Hungarian survey in 2014) questionnaire, which consists of four modules on (a) health status, (b) health care use, (c) health determinants, and (d) socio-economic variables. In these modules, the following topics are covered: (a) self-perceived health, chronic diseases known by the respondents, limitation in activities, and mental health, (b) use of different types of health care services, including hospitalizations, consultations, preventive services, and medications, and unmet needs for health care, and (c) smoking and alcohol consumption, physical activity, and dietary habits and additional background variables on demographics and socio-economic status such as sex, age, living conditions, education, income, and employment [40].

The EHIS questionnaire was extended with some additional groups of questions validated in previous surveys [41,42,43,44,45,46].

#### 2.3.2. II. Physical Examinations

Anthropometric (weight, height and waist circumference) and blood pressure (BP) measurements were obtained for each survey participant by using the European Health Examination Survey protocol [47]. Body mass index (BMI) was calculated by using the following formula: BMI = weight (kg)/height (m)^2^. Routine physical examination was extended to define visual acuity and physical/cardiovascular fitness.

#### 2.3.3. III. Laboratory Examinations

After overnight fasting, native (5 mL) and EDTA-anticoagulated whole blood samples (2 × 3 mL) were taken for laboratory tests and DNA extraction. For biochemical investigations, serum and plasma samples were separated by centrifugation at 3000 rpm for 10 min and used immediately or kept at −80 °C until analysis. DNA preparation was carried out from EDTA-anticoagulated blood samples on the day of sample collection.

In the Department of Laboratory Medicine of the University of Debrecen, total cholesterol (C), HDL-C, LDL-C, triglycerides (TG), glucose (Glu), creatinine, uric acid, C-reactive protein (CRP), apolipoprotein A1 (ApoA1), apolipoprotein B100 (ApoB100) concentrations, alanine aminotransferase (ALT), aspartate aminotransferase (AST), gamma-glutamyl-transferase (GGT), alkaline phosphatase (ALP) activities, folic acid, Haemoglobin A1c, and insulin concentrations were determined.

From plasma samples lipid profiling and on DNA samples genotyping analyses are in progress.

### 2.4. Creation of the Database

The data management process was coordinated by the Public Health Research Institute of the University of Debrecen. All validation rules (skip, range, and consistency checks) provided previously to the EHIS 2 by Eurostat were strictly followed and processed [40]. Data obtained from interviewer-assisted questionnaires as well as from physical and laboratory examinations were input into an Excel spreadsheet type database with the type of data as column headers and anonymized participants in the rows.

Data for the determination of the prevalence of MetS and its components and the calculation of surrogate indices to estimate the prevalence of IR were extracted from the database of the complex survey. Namely, the results of waist circumference and blood pressure measurements, fasting TG, HDL-C, Glu, and insulin concentrations, as well as antihypertensive, antidiabetic therapies, and specific treatments for lipid abnormalities were sorted.

### 2.5. Determination of the Prevalence of Metabolic Syndrome and Its Components

The prevalence rates of MetS and that of its components were defined by accepting the IDF definition, i.e., someone was considered to have metabolic syndrome if he or she had central adiposity (waist circumference: ≥94 cm for men and ≥80 cm for women—for Europid population) combined with two or more of the following four factors: (1) raised concentration of triglycerides (≥1.7 mmol/L) or specific treatment for this lipid abnormality; (2) reduced concentration of HDL cholesterol: (<1.03 mmol/L in men and <1.29 mmol/L in women) or specific treatment for this lipid abnormality; (3) raised blood pressure (systolic blood pressure ≥ 130 mmHg or diastolic blood pressure ≥ 85 mmHg) or treatment of previously diagnosed hypertension; and (4) raised fasting plasma glucose concentration (≥5.6 mmol/L) or previously diagnosed type 2 diabetes [48].

### 2.6. Insulin Sensitivity/Resistance Indices Calculated by Using Fasting Serum/Plasma Concentrations of Insulin, Glucose, HDL-C and Triglycerides

The method accepted as the gold standard for investigating and quantifying insulin resistance, the hyperinsulinaemic euglycaemic clamp technique [49], is not applicable in population-based surveys (it involves intravenous infusion of insulin, frequent blood sampling over a 2-h period, and continuous adjustment of a glucose infusion) [50]. Thus, indirect indices are used in population studies because of their technical simplicity (see reviewed by [51]). To estimate the risk of insulin resistance, surrogate indices, such as the homeostasis model assessment of insulin resistance, HOMA-IR [52], defined as (glucose (mmol/L) × insulin level (mIU/L)/22.5), the quantitative insulin sensitivity check index, QUICKI [53], defined as (1 − (log glucose + log insulin)), and the McAuley index [54] as defined (exp ((2.63–0.28 ln [insulin (IU/mL)] − 0.31 ln [TG (mmol/L)])) were calculated. In addition, the TyG index has been proposed as a useful surrogate measure of insulin resistance in healthy adults [55]; it is calculated by using the equation ln[triglycerides (mmol/L) × glucose (mmol/L)/2] and TG/HDL-C ratio [56] and was demonstrated to be a good indicator of IR in numerous studies (see discussed in [57]).

#### 2.6.1. Determination of the Cut-Off Values of Surrogate Measures for Insulin Resistance

Surrogate indices were calculated for each participant in both study groups. To estimate the cut-off values of the surrogate measures of insulin sensitivity for discriminating metabolic syndrome, the receiver operating characteristic (ROC) curve was applied. Youden’s method [58] was used to find an optimal cut-off point on the ROC curves to optimize the sensitivity and the specificity of each IR index. The index was calculated for all points of the ROC curves, and the maximum value of the index was used as a criterion for selecting the optimum cut-off point. To characterize the indicative power of different biochemical parameters and surrogate indices for MetS/IR, area under the curve (AUC) calculations were carried out. Analyses were performed using SPSS software version 26.0 (IBM, Armonk, NY, USA) [59].

#### 2.6.2. Determination of the Prevalence of IR by Using Different Surrogate Indices

The prevalence of IR was defined for the HG and HR populations by using population-specific cut-off points and cut-off values defined in combined populations for the HOMA-IR, QUICKI, McAuley index, TG/HDL-C ratio, and TyG index. The prevalence of IR was defined as the proportion (%) of the populations with surrogate index values higher than or equal to the cut-off points by age and sex for both populations by using the HOMA-IR, McAuley, and TyG indices.

### 2.7. Statistical Analysis

The age- and sex-specific prevalence of the different measures was calculated. Subjects were categorized by age as follows: 20–34, 35–49, and 50–64 years. The exact 95% confidence interval (95% CI) was computed for all point estimates. SPSS software version 26.0 was used for statistical analyses. Differences in prevalence were evaluated by the 95% CI presented. The difference was considered as significant at *p* < 0.05.

### 2.8. Ethical Statement

All subjects gave their informed consent for inclusion before they participated in the study. The study was conducted in accordance with the Declaration of Helsinki, and the protocol was approved by the Ethics Committee of the Hungarian Scientific Council on Health (61327-2017/EKU).

## 3. Results

**Figure 2:**
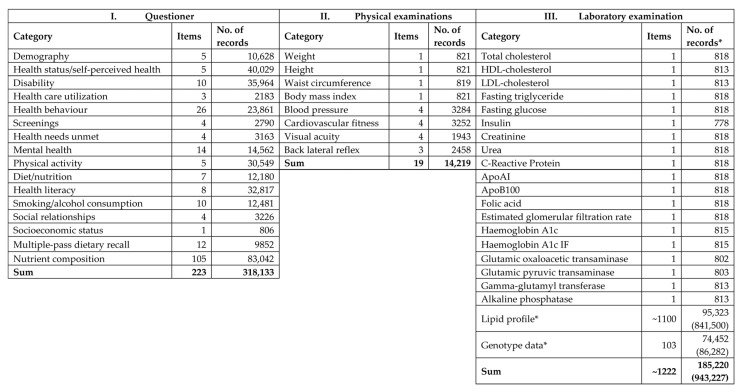
Content of the database by three pillars of the survey. *: available data (expected data).

Because the sample number planned was 500 for both populations, the response rates for completing the questionnaires were 83.4% (*n* = 417) in the Hungarian general population and 83.0% (*n* = 415) in the Roma population. The flowchart of the process of sample management (Figure 1) shows the reasons why 20 subjects from the HG and 47 from the HR populations were excluded. Ultimately, in the database created, more than half a million datapoints were entered (Figure 2) from the full records of 765 participants (397 from the HG and 368 from the HR sample).

### 3.1. Demographic and Anthropometric Characteristics of the Samples

Differences in demographic and anthropometric characteristics were observed between the HG and HR population samples (Table 1). The representation of females was significantly higher in the HR (73.6%) than in the HG (55.4%) population. Although the age distribution of the HR sample was slightly shifted towards the youngest age group (20–34 years), no significant difference could be observed between the age distribution of the HG and HR samples. The average ages in the HG and HR populations were 44.2 and 42.8 years, respectively. Both the average weight and height were significantly higher in the HG population (77.8 kg vs. 71.9 kg and 168.7 cm vs. 161.2 cm, respectively). Thus, the average BMI values did not differ significantly.

### 3.2. The Prevalence of MetS and Its Components in the Study Populations

The prevalence of metabolic syndrome was very high in both study populations (HG 39.8%, HR 44.0%), especially among Roma males (46.4%), but no significant difference could be shown between the two groups in either females or males (Table 2). Regarding MetS components, the prevalence of central obesity was the highest (HG 75.6%, HR 73.4%), followed by that of increased blood pressure or treated hypertension (HG 57.2%, HR 54.1%). Significant differences in the prevalence of reduced HDL-C concentration could be detected in both sexes, as well as at the population level between HG and HR groups due to the extremely high prevalence among Roma compared to the HG population (56% vs. 36.3% at population level, 47.4% vs. 32.2% for males, and 59.0% vs. 39.6% for females).

### 3.3. Findings Used to Estimate the Risk of IR for MetS

Table 3 shows that regarding data defined by the questionnaire-based survey, as well as by laboratory and physical examinations and used in IR and MetS calculations, significant differences were observed in the average HDL-C concentration and systolic blood pressure; both values were significantly lower in the HR population than in the HG population (1.26 mmol/L vs. 1.37 mmol/L, 123.7 mmHg vs. 126.8 mmHg, respectively). In the case of these two parameters, significant differences were observed in subgroups without and with MetS in harmony with our previous findings on the genetic background of the high prevalence of low HDL-C concentration [20] and low prevalence of hypertension [60] among Roma in comparison with the HG population. The prevalence rates of antidiabetic and lipid-lowering therapies were significantly higher among the HR population (11.1% vs. 6.1%, 11.7% vs. 6.8%, respectively), while no significant difference was found in any of the other parameters between the study groups.

Average surrogate indices calculated for the two population groups did not differ significantly. In harmony with findings on the mean TG and HDL-C concentration values, a significant difference was observed in the case of the TG/HDL-C ratio being less favourable for the HR than for the HG population (1.49 vs. 1.36, *p* = 0.033) (Table 4).

**Figure 3:**
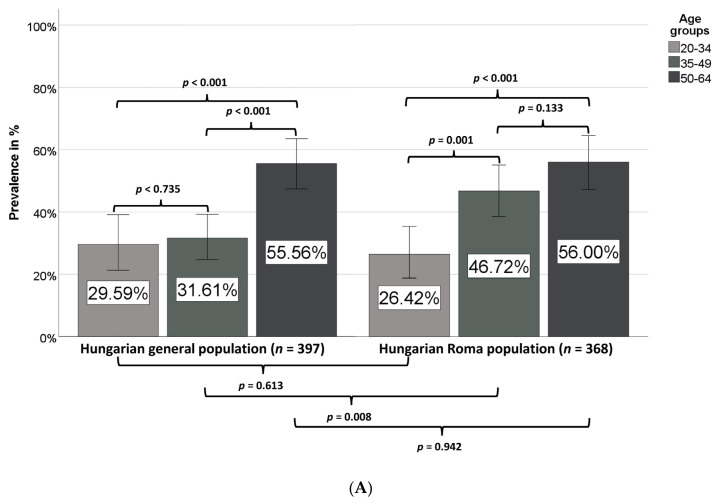

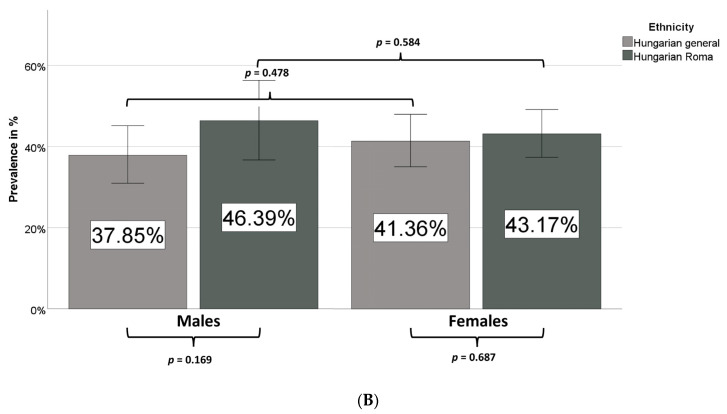
Prevalence of metabolic syndrome in different age groups (**A**) and sexes (**B**) in the Hungarian general and Hungarian Roma populations.

The prevalence of MetS strongly increased by age in both the HG and HR populations (Figure 3), but while it was only slightly higher in the 35–49 years age group than in the 20–34 years age group in the HG population (31.6% vs. 29.6%), a very marked increase in prevalence was observed in the 35–49 years age group in comparison with the 20–34 years age group in the HR population (46.7% vs. 26.4%).

While in the case of the Hungarian general population the prevalence of MetS is significantly increased in the age group of 50–64 years, a significant increase in MetS prevalence is observed in the age group of 35–49 years among Roma in comparison with the age group of 20–34 years (Figure 3A). No statistically significant difference was observed between males or females in the two groups (Figure 3B).

### 3.4. Determination of Cut-Off Points for IR Surrogate Indices

As it was shown (Table 3 and Table 4) that the mean values of both biochemical parameters and surrogate indices strongly differed between subgroups without and with MetS in both study populations and as—except for HDL-C mean values—no significant difference could be detected between the HG and HR population subgroups (without and with MetS), the sensitivity, specificity, Youden’s index and cut-off points for each surrogate measure (biochemical parameters and indices) were defined not only for HG and HR but also for the combined population.

Among the biochemical variables (Table 5A), Youden’s index was the highest (0.562) for fasting TG at 1.59 mmol/L in the combined population, with 71% sensitivity and 85% specificity to indicate MetS/IR. Among Roma, Youden’s index for fasting TG was as high as 0.605, with low sensitivity (67%) but relatively high specificity (93%). Concerning surrogate indices (Table 5B), cut-off values were determined by using Youden’s index, with results varying between 0.399 and 0.615. The cut-off values of HOMA-IR (as 2.32) and QUICKI (0.34) could be defined with relatively low sensitivity and specificity (71% and 69% for both indices, respectively) in comparison with those of the McAuley index (5.99) and TG/HDL-C ratio (1.27), with similar sensitivity (70% and 73%, respectively) but with much higher specificity (83% and 84%, respectively). The cut-off value of 4.69 with 77% sensitivity and 84% specificity for the TyG index seems to be the surrogate index with the most favourable prognostic/diagnostic performance in our study. On the basis of the results obtained by AUC analyses, the predictive power of the TyG index was found to be the highest for MetS/IR. These results are in harmony with the finding that the highest AUC value was obtained for fasting TG among the biochemical parameters used for the surrogate index calculations (Table 5).

### 3.5. Prevalence of IR in the Hungarian General and Hungarian Roma Populations

The prevalence values defined varied in a relatively wide range (38.8–48.1% for HG and 40.5–53.2% for HR) by using different population-specific cut-off values and in a slightly narrower range (37.8–47.6% for HG and 40.5–47.8%) if cut-off values defined on the combined population were applied (Table 6). Except for the IR prevalence values defined by using the McAuley index in separate populations (42.6% for HG and 53.3% for HR), no significant difference was observed between the two groups. The 53.3% IR prevalence for the HR population defined by the McAuley index was exceptionally high, which was not comparable with the results obtained by using any other surrogate indices. If the McAuley index cut-off value defined on the combined population was used, the prevalence rates could be defined as 37.8% for the HG and 40.8% for the HR population. By using cut-off values obtained on the combined population, the highest prevalence values were determined by the HOMA-IR and QUICKI (47.6% for HG and 47.8% for HR, 47.1% for HG and 47.8% for HR, respectively), while the lowest prevalence values could be defined by the TG/HDL-C ratio (39.3% for HG and 40.5% for HR). The prevalence values defined by using TyG index cut-off points were barely different if the population-specific cut-off points or the cut-off points defined on combined population were used (42.3% for HG and 40.8% for HR, 42.3% for HG and 40.5% for HR, respectively).

The prevalence values by age did not differ significantly between the two populations (Table 7A), except for the IR prevalence values defined by using the HOMA-IR in the age group of 50–64 years (60.4% for HG and 45.6% for HR, *p* = 0.015). In contrast to the expectation, increasing prevalence by age could not be detected by either the HOMA-IR (consequently by QUICKI) or the McAuley indices in the HR population; the *p* for trend values showed significant increases in the IR prevalence in both groups only if the TyG index was used for calculation.

HOMA-IR-based prevalence values showed no significant differences between the two groups by sex and ethnicity. Calculations using the McAuley index indicated a higher IR prevalence in both sexes among Roma (Table 7B), but the IR prevalence values did not differ significantly between sexes in either the HG or the HR populations. TyG index-based estimates did not indicate a significant difference by ethnicity but clearly showed a higher IR prevalence among men in both groups.

As the TyG index showed the greatest power for predicting IR to estimate MetS risk in both populations, the IR/MS prevalence was defined by using TyG and was found to be as high as 42.3% and 40.5% in the HG and HR populations, respectively.

## 4. Discussion

In our present study, a unique database was created in which questionnaire-based survey results, physical examination and laboratory data obtained in a complex survey on participants from the general and Roma populations living in North-East Hungary were collected. The database consists of more than half a million data points available for comparative descriptive and association analyses. It is a very valuable resource not only to examine the prevalence of unhealthy conditions and susceptibility to different diseases, but also to explore the environmental and genetic factors contributing to their development. In addition to the more precise characterization of the health status and disease burden/risk of the Hungarian general population, the findings can also help to understand the unfavourable health status of Roma people. Roma—independently, where they live—represent the most vulnerable ethnic group of the European population [15,16,61], with a continuously increasing representation [62,63]. It has been thoroughly demonstrated that Roma people are overrepresented in marginalized communities and live in unhealthy environments, and their health behaviour is significantly less favourable than that of the non-Roma population [38,64,65,66]. The health of Roma can be considered as global ill health [1], based not only on the burden of rare and infectious diseases [4,5], but also on chronic non-communicable diseases of high public health importance, such as cardiometabolic diseases, which are significantly higher among them than in the majority population [7,8,9,11,12,13,67]. In addition, the access of Roma people to health care is also impaired, resulting in a broad spectrum of unmet health needs [68,69,70,71]. Although our previous studies strongly suggest increased genetic susceptibility to certain cardiometabolic diseases through leading to high prevalence of decreased HDL-C level and increased risk of thrombosis among Roma [19,20], we have found no difference between the Hungarian general and Roma populations in genetic risk for diabetes and obesity [72,73]. The admixture of Roma with West Eurasians before their exodus and during their migration period besides their admixture with Europeans is a well-known fact, but using identity-by-descent segment (IDS) analysis Melegh et al. [74] demonstrated that Roma are closer related to European populations. Central and Eastern European populations show a significantly higher share in the European ancestry of Roma than other regions of Europe. Average shared IBD segment length of Roma with Central European populations was 0.355 Mb.

Improvements in their health status would make an important contribution to the economic progress of the countries where they live with increasingly representation. The metaphor from Thomas Reid’s Essays on the Intellectual Powers of Man, published in 1785 [75], of “a chain is only as strong as its weakest link”, is also very relevant for Roma inclusion initiatives.

In our present study, as a demonstration of the usefulness of the database we have developed, the prevalence and development dynamics of insulin resistance and metabolic syndrome in the Hungarian Roma population accumulated in the North-East part of the country were investigated in comparison with these characteristics in the Hungarian general population.

In epidemiological studies, surrogate indices using anthropometric (waist circumference and blood pressure measurements) and biochemical variables, such as fasting insulin, glucose, and TG and HDL-C levels, are widely used [51,76]. While there is general agreement among the authors of myriad publications on the predictive power of different surrogate markers of IR, namely, that they adequately indicate IR and are good predictors of MetS, especially when using the International Diabetes Foundation classification, studies on different populations found different indices to be “the best”. In a Korean sample [77] and in the Cyprus Study [78], the McAuley index showed the best accuracy, while in another Korean survey [59], in a Chinese study [79], and in a White European cohort (Vascular-Metabolic CUN cohort) in Spain, the TyG index was found to have the best sensitivity and specificity to detect MetS/diabetes [80]. In a comparative study carried out by Kahn et al. [81]—in harmony with findings of initial studies [47,82] that identified TyG as a tangible marker for metabolic syndrome and underlying insulin resistance—the TyG index, with the highest AUC in comparison to fasting glucose, TG, LDL-C, non-HDL-C, and the HOMA-IR, was found to be the most efficient marker for diagnosing MetS. It was presented by the authors that the HOMA-IR showed the lowest AUC in comparison to other evaluated markers [81].

In our present study, the prevalence of MetS was very high in both study populations (HG 39.8%, HR 44.0%), especially among Roma males (46.4%), but no statistically significant difference could be shown between the two groups for either females or males. By defining the MetS prevalence by age groups, we could show that MetS arises earlier among Roma than in the HG population; while there is no significant difference in MetS prevalence between HG and HR populations in the 20–34 years age group (29.6% and 26.4%, respectively), it is significantly higher in the HR population than in the HG population in the 35–49 years age group (46.7% vs. 31.6%), i.e., in comparison with the prevalence values for the age group of 20–34 years a significant increase in MetS prevalence can be observed in the age group of 35–49 years among Roma, while in case of the HG population it can be detected only in the older age group (50–64 years). Regarding the prevalence of different MetS components, a significant difference could be detected only in the frequency of reduced HDL-C concentration, which was as high as 56% (47.4% for males and 59.0% for females) among Roma (in the HG population overall and in HG males and females, these values were 36.3%, 32.2%, and 39.6%, respectively). This finding is in accordance with previous findings on the high prevalence of participants with reduced HDL-C level not only among Hungarian Roma [83] but also in the Slovakian Roma population [48]. In our previous study, we confirmed that a genetic background exists behind this phenomenon [20]. The prevalence of central obesity was extremely high in both groups (HG 75.6%, HR 73.4%), and that of increased blood pressure or treated hypertension was also very high (HG 57.2%, HR 54.1%). It is worth mentioning that in comparison with the prevalence of hypertension, the prevalence of patients with antihypertensive medication was as low as 28.5% in the HG population and not significantly higher among Roma (31.5%), indicating a severe risk for hypertension underdiagnosis and undertreatment in both groups.

To define the prevalence of insulin resistance, we have determined the cut-off values of the five surrogate IR indices (HOMA-IR, QUICKI, McAuley, TG/HDL-C, and TyG) most frequently used in clinical and population studies for the development of MetS in separate and combined populations. Since no significant difference could be detected between the results obtained in separate and combined populations, cut-off values defined on the combined population were used for risk prediction. As the TyG had the best indicative power (cut-off value of 4.69 with 77% sensitivity and 84% specificity) for predicting IR to estimate MetS risk in both populations, the IR prevalence was defined by using TyG and was found to be as high as 42.3% and 40.5% in the HG and HR populations, respectively. The prevalence of IR is consistent with that of MetS—slightly higher in the HG and slightly lower in the HR population, but the differences were not significant. It is important to mention that the increase in MetS prevalence by age as a significant trend in both groups could be detected only by the TyG index at the level of IR. Consequently, we can propose the TyG index instead of other surrogate indices to estimate the risk of IR as having indicative power for MetS development.

The only survey that is comparable with our present one—in which data were collected via questionnaire, anthropometric measures and analysed blood and urine samples—was named HepaMeta and was carried out in 2011 in Eastern Slovakia to define the prevalence of Hepatitis B/C and metabolic syndrome in populations living in segregated Roma colonies in comparison with the general Slovakian population [84]. In the HepaMeta study, Roma and non-Roma participants aged between 18 and 55 years were enrolled, and MetS was found to be more common in the Roma than in the general population (29.6% vs. 20.1%, respectively, *p* < 0.0001) [85]. Although the Slovakian researchers used the same (IDF) criteria to identify individuals with MetS, their findings strongly differed from ours. The main reasons for the difference in data on MetS prevalence might be as follows:

(a) The Slovakian researchers recruited participants aged 18–55 years (i.e., their study population was roughly a decade younger than ours), which may result in the significantly higher MetS prevalence among Roma than non-Roma populations observed in the Slovakian study. As we show in our present study, the age of MetS onset is significantly lower among Roma (the MetS prevalence in the age group of 35–49 years is 46.7% among Hungarian Roma and 31.6% in the HG population, while the prevalence in the 50–64 year age group is almost the same in the two populations).

(b) In addition, the prevalence of central obesity is significantly higher among Slovakian Roma than in the majority population (58.9% vs. 45.8%), while no significant difference was observed between the two populations in our study. In addition, the prevalence of reduced HDL-C was found to be double among Roma in comparison with their non-Roma counterparts (70% in the Roma population and 34.9% among non-Roma). In our study, the relevant prevalence values were 56% vs. 36.3% (i.e., the ratio is 1.54).

(c) The Slovakian study was carried out in 2011, while our present survey was conducted in 2018. In our previous study, which was performed in a representative random sample of the Roma population aged 20–64 years living in segregated colonies in North-East Hungary in 2011 (i.e., in the very same year as the Slovakian HepaMeta study), the prevalence of central obesity was much lower in the Roma population both in absolute value and in comparison with the general population [83]. Consequently, the prevalence of MetS was also much lower in the sample from 2011 than in our present study in both the Roma and the general populations (36.4% and 35% in 2011 and 44% and 39.8% in 2018, respectively). This finding is consistent with the results of our comparative analysis on data of two Roma surveys carried out before and after the Decade of Roma Inclusion (in 2004 and 2015), in which we demonstrated that obesity among Roma (especially in the younger age groups) became significantly more frequent during that decade [8].

The main strength of our present study is that, as an outcome of a complex health (behaviour and examination) survey among Roma living in colonies in North-East Hungary and their surrounding general Hungarian population, a very valuable database was created that can be used in comparative and association studies. By using selected data, the prevalence of insulin resistance was first defined in Roma and the general Hungarian populations by comparing the results obtained after defining the cut-off values for different surrogate indices and identifying the index with the best indicative power to estimate the risk of the development of metabolic syndrome.

The major limitation of the study is that females are overrepresented in the Roma sample. This cross-sectional survey was based on randomly selected households, and in many households, only women were home during the day when most visits took place, while men had travelled at least locally for public work. The Hungarian government quadrupled the budget for public works between 2010 and 2015 for all Hungarian municipalities. This is especially relevant for villages in the North-eastern region of Hungary, where segregated Roma settlements are concentrated. The majority of workers participating in the programme are men from deprived Roma communities. In addition, elderly (aged over 65 years) citizens are not represented in the sample. As determined in our previous Roma surveys [8,64,83], the representation of people over 65 years was as low as 3–4%. Thus, the size of the strata 65-X was too small to make reliable conclusions for this subgroup of the population. Among the limitations it is also worth mentioning that the data obtained among Roma were compared to the data on the Hungarian general population, one of the most obese adult (15+ years) populations (with 62.3% prevalence of overweight/obesity) among OECD countries [8,62,64,83], which may lead to an underestimation of the difference between the two populations.

## 5. Conclusions

The prevalence of IR, as well as that of MetS, which are the most robust indicators for the risk of cardiometabolic diseases, do not differ significantly between the Hungarian Roma population and the Hungarian general population. These values are almost equally unfavourable for both groups, but the premature mortality of Roma is much higher, as indicated by the very low representation of the 65-X age group among Roma in our previous studies. The findings clearly indicate that the access of Roma people to health services (including both preventive and curative services) should be closely evaluated and inequities urgently targeted by effective interventions.

## Figures and Tables

**Table 1:** Anthropometric and demographic characteristics of the study populations.

		Hungarian General Population (*n* = 397)	Hungarian Roma Population (*n* = 368)	*p*-Value
		Prevalence % (*n*)	Prevalence % (*n*)	
Sex	Male	44.58 (177)	26.36 (97)	<0.001
	Female	55.42 (220)	73.64 (271)	
Age groups	20–34	24.69 (98)	28.80 (106)	0.434
	35–49	39.04 (155)	37.23 (137)	
	50–64	36.27 (144)	33.97 (125)	
		**Mean (95% CI)**	**Mean (95% CI)**	***p*-Value**
Age (years)		44.18 (42.97–45.38)	42.82 (41.57–44.07)	0.123
Weight (kg)		77.80 (76.12–79.47)	71.91 (69.93–73.89)	<0.001
Height (cm)		168.73 (167.79–169.68)	161.15 (160.20–162.11)	<0.001
BMI (kg/m^2^)		27.27 (26.74–27.81)	27.61 (26.90–28.31)	0.918

95% CI: 95% confidence interval.

**Table 2:** Prevalence of metabolic syndrome and its components in the Hungarian general (HG) and Hungarian Roma (HR) populations by ethnicity and sex.

Metabolic Syndrome and Its Components	Hungarian General Population (*n* = 397)	Hungarian Roma Population (*n* = 368)	*p*-Value	Males in the HG Population (*n* = 177)	Males in the HR Population (*n* = 97)	*p*-Value	Females in the HG Population (*n* = 220)	Females in the HR Population (*n* = 271)	*p*-Value
	Prevalence % (95% CI)	Prevalence % (95% CI)		Prevalence % (95% CI)	Prevalence % (95% CI)		Prevalence % (95% CI)	Prevalence % (95% CI)	
Central obesity	75.56 (70.11–78.65)	73.37 (68.68–77.69)	0.719	64.41 (57.17–71.18)	58.76 (48.83–68.18)	0.356	82.73 (77.32–87.28)	78.60 (73.43–83.16)	0.251
Raised blood pressure or treated hypertension	57.18 (52.27–61.98)	54.08 (48.97–59.12)	0.378	60.45 (53.13–67.44)	57.73 (47.79–67.22)	0.661	54.55 (47.94–61.03)	52.77 (46.82–58.66)	0.694
Raised fasting plasma glucose concentration or previously diagnosed diabetes mellitus	25.19 (21.11–29.63)	23.91 (19.77–28.46)	0.666	27.12 (20.98–34.00)	31.96 (23.31–41.66)	0.398	23.64 (18.39–29.57)	21.03 (16.50–26.18)	0.490
Raised triglyceride level or treated lipid disorder	37.28 (32.63–42.11)	37.77 (32.93–42.81)	0.767	42.94 (35.81–50.30)	51.55 (41.68–61.32)	0.172	32.73 (26.78–39.12)	32.84 (27.46–38.59)	0.979
Reduced HDL cholesterol level or treated lipid disorder	36.27 (31.66–41.09)	55.98 (50.88–60.99)	<0.001	32.20 (25.65–39.33)	47.42 (37.68–57.31)	0.013	39.55 (33.26–46.11)	59.04 (53.12–64.77)	<0.001
Metabolic syndrome	39.80 (35.07–44.67)	44.02 (39.01–49.12)	0.232	37.85 (30.95–45.15)	46.39 (36.70–56.30)	0.169	41.36 (35.00–47.95)	43.17 (37.37–49.12)	0.687

Significant differences are highlighted in grey. 95% CI: 95% confidence interval.

**Table 3:** Mean value of biochemical and physical parameters used to estimate the risk of insulin resistance and metabolic syndrome in the Hungarian general (HG) and Hungarian Roma (HR) populations furthermore obtained on samples without and with metabolic syndrome.

				Without Mets			With Mets		
	HG (*n* = 397)	HR (*n* = 368)	*p*-Value	HG (*n* = 239)	HR (*n* = 206)	*p*-Value	HG (*n* = 158)	HR (*n* = 162)	*p*-Value
	Mean			Mean			Mean		
Fasting insulin (mU/L)	15.82	16.67	0.892	11.02	11.27	0.371	23.10	23.54	0.736
Fasting glucose (mmol/L)	5.25	5.10	0.105	4.77	4.63	0.158	5.98	5.70	0.177
Fasting TG (mmol/L)	1.58	1.61	0.375	1.18	1.12	0.941	2.19	2.23	0.386
HDL-C (mmol/L)	1.37	1.26	<0.001	1.49	1.37	0.001	1.19	1.12	0.006
Waist circumference (cm)	96.03	94.78	0.286	89.6	86.9	0.008	105.8	104.8	0.580
Systolic blood pressure (mmHg)	126.75	123.71	< 0.001	122.4	119.3	0.007	133.4	129.3	0.001
Diastolic blood pressure (mmHg)	78.82	79.63	0.600	76.9	76.6	0.469	81.7	83.4	0.229
Prevalence of antihypertensive treatment (%)	28.46	31.52	0.356	12.97	13.11	0.966	51.90	54.94	0.586
Prevalence of antidiabetic treatment (%)	6.05	11.14	0.012	0.84	1.94	0.314	13.92	22.84	0.040
Prevalence of lipid lowering therapy (%)	6.80	11.68	0.019	1.67	1.46	0.845	14.56	24.69	0.023

Significant differences are highlighted in grey.

**Table 4:** Average surrogate index values calculated for the Hungarian general (HG) and Hungarian Roma (HR) populations.

Indices				Without MetS			With MetS		
	HG (*n* = 397)	HR (*n* = 368)	*p*-Value	HG (*n* = 239)	HR (*n* = 206)	*p*-Value	HG (*n* = 158)	HR (*n* = 162)	*p*-Value
	Mean			Mean			Mean		
HOMA-IR	4.07	4.32	0.722	2.51	2.47	0.321	6.44	6.68	0.568
QUICKI	0.34	0.35	0.722	0.36	0.37	0.320	0.32	0.32	0.568
McAuley index	6.96	7.00	0.746	7.94	8.20	0.375	5.48	5.47	0.881
TG/HDL-C ratio	1.36	1.49	0.033	0.90	0.90	0.110	2.05	2.24	0.085
TyG index	4.64	4.64	0.871	4.47	4.45	0.743	4.88	4.88	0.978

Significant differences are highlighted in grey.

**Table 5:** The cut-off points and their sensitivity, specificity and Youden’s index calculated on the basis of area under curve for each surrogate measure—as biochemical parameters (**A**) and indices (**B**)—in the Hungarian general and Hungarian Roma populations and in the combined study population.

**A**												
**Variables**	**Hungarian General Population (*n* = 397)**				**Hungarian Roma Population (*n* = 368)**				**Combined Population (*n* = 765)**			
	**Cop**	**Sens./Spec.**	**YI**	**AUC**	**Cop**	**Sens./Spec.**	**YI**	**AUC**	**Cop**	**Sens./Spec.**	**YI**	**AUC**
Fasting insulin (mU/L)	12.480	0.614/0.749	0.363	0.735	10.100	0.728/0.650	0.379	0.726	11.855	0.634/0.712	0.347	0.730
Fasting glucose (mmol/L)	5.350	0.506/0.828	0.335	0.696	5.550	0.364/0.927	0.291	0.674	5.350	0.450/0.852	0.302	0.684
Fasting TG (mmol/L)	1.590	0.709/0.828	0.537	0.826	1.750	0.673/0.932	0.605	0.857	1.590	0.713/0.849	0.562	0.841
HDL-C (mmol/L)	1.195	0.620/0.808	0.428	0.745	1.210	0.753/0.675	0.428	0.744	1.195	0.675/0.753	0.428	0.743
**B**												
**Indices**	**Hungarian General Population (*n* = 397)**				**Hungarian Roma Population (*n* = 368)**				**Combined Population (*n* = 765)**			
	**Cop**	**Sens./Spec.**	**YI**	**AUC**	**Cop**	**Sens./Spec.**	**YI**	**AUC**	**Cop**	**Sens./Spec.**	**YI**	**AUC**
HOMA-IR	2.291	0.747/0.695	0.441	0.763	2.224	0.710/0.660	0.370	0.744	2.320	0.709/0.690	0.399	0.753
QUICKI	0.337	0.747/0.695	0.441	0.763	0.338	0.710/0.660	0.370	0.744	0.336	0.709/0.690	0.399	0.753
McAuley index	6.297	0.741/0.782	0.523	0.825	6.768	0.833/0.704	0.537	0.828	5.989	0.697/0.827	0.524	0.827
TG/HDL-C ratio	1.304	0.722/0.833	0.554	0.831	1.274	0.747/0.864	0.611	0.855	1.274	0.734/0.843	0.574	0.843
TyG index	4.694	0.791/0.820	0.611	0.858	4.685	0.759/0.869	0.628	0.867	4.694	0.772/0.843	0.615	0.862

Cop: cut-off point; Sens.: sensitivity; Spec.: specificity; YI: Youden’s index (sensitivity + specificity-1); AUC: area under curve.

**Table 6:** Prevalence of insulin resistance (IR) and number of persons with IR in the Hungarian general and Hungarian Roma populations.

Indices	Prevalence of Insulin Resistance (%) Based on the Cut-Off Points Identified in the Study Populations (95% CI)		*p*-Value	Prevalence of Insulin Resistance (%) Based on the Cut-Off Points Identified in the Combined Population (95% CI)		*p*-Value
	Hungarian General Population (*n* = 397)	Hungarian Roma Population (*n* = 368)		Hungarian General Population (*n* = 397)	Hungarian Roma Population (*n* = 368)	
HOMA-IR	48.11 (43.22–53.02) *n* = 191	49.73 (44.64–54.82) *n* = 185	0.550	47.61 (42.73–52.52) *n* = 189	47.83 (42.76–52.93) *n* = 176	0.952
QUICKI	48.11 (43.22–53.02) *n* = 191	49.46 (44.37–54.55) *n* = 182	0.710	47.10 (42.23–52.02) *n* = 187	47.83 (42.76–52.93) *n* = 176	0.841
McAuley index	42.57 (37.77–47.47) *n* = 169	53.26 (48.15–58.32) *n* = 196	0.003	37.78 (33.12–42.63) *n* = 150	40.76 (35.83–45.84) *n* = 150	0.399
TG/HDL-C ratio	38.79 (34.09–43.65) *n* = 154	40.49 (35.56–45.56) *n* = 149	0.631	39.29 (34.58–44.16) *n* = 156	40.49 (35.56–45.56) *n* = 149	0.736
TyG index	42.32 (37.53–47.22) *n* = 168	40.76 (35.83–45.84) *n* = 150	0.663	42.32 (37.53–47.22) *n* = 168	40.49 (35.56–45.56) *n* = 149	0.608

95% CI: 95% confidence interval; *n*: sample number.

**Table 7:** Prevalence of insulin resistance based on the homeostasis model assessment of insulin resistance (HOMA-IR) index, McAuley index, and TyG index (calculated on the combined population) in the Hungarian general (HG, *n* = 397) and Hungarian Roma (HR, *n* = 368) populations by age group in years (**A**) and sex (**B**).

**A**									
	**HOMA-IR**			**McAuley Index**			**TyG Index**		
**Age Groups**	**HG**	**HR**	***p*-Value**	**HG**	**HR**	***p*-Value**	**HG**	**HR**	***p*-Value**
	**Prevalence %; *n***			**Prevalence %; *n***			**Prevalence %; *n***		
20–34	44.90; *n* = 90	50.00; *n* = 53	0.466	32.65; *n* = 32	40.57; *n* = 43	0.242	32.65; *n* = 32	30.19; *n* = 32	0.705
35–49	37.42; *n* = 58	48.18; *n* = 66	0.064	33.55; *n* = 52	41.61; *n* = 57	0.155	38.06; *n* = 59	40.88; *n* = 56	0.624
50–64	60.42; *n* = 87	45.60; *n* = 57	0.015	45.83; *n* = 66	40.00; *n* = 50	0.335	53.47; *n* = 77	48.80; *n* = 61	0.444
***p* for trend**	0.004	0.502		0.044	0.965		0.002	0.016	
**B**									
	**HOMA-IR**			**McAuley Index**			**TyG Index**		
**Sex**	**HG**	**HR**	***p* for Ethnicity**	**HG**	**HR**	***p* for Ethnicity**	**HG**	**HR**	***p* for Ethnicity**
	**Prevalence %; *n***			**Prevalence %; *n***			**Prevalence %; *n***		
Males	49.72; *n* = 88	50.52; *n* = 49	0.899	44.07; *n* = 78	49.48; *n* = 48	0.021	49.15; *n* = 87	51.55; *n* = 50	0.705
Females	45.91; *n* = 101	46.86; *n* = 127	0.833	32.70; *n* = 72	37.64; *n* = 102	0.042	36.82; *n* = 81	36.53; *n* = 99	0.948
***p* for sex**	0.450	0.537		0.390	0.258		0.013	0.010	
